# microRNA profiling in lung tissue and bronchoalveolar lavage of cigarette smoke-exposed mice and in COPD patients: a translational approach

**DOI:** 10.1038/s41598-017-13265-8

**Published:** 2017-10-09

**Authors:** Griet Conickx, Francisco Avila Cobos, Maarten van den Berge, Alen Faiz, Wim Timens, Pieter S. Hiemstra, Guy F. Joos, Guy G. Brusselle, Pieter Mestdagh, Ken R. Bracke

**Affiliations:** 1 Laboratory for Translational Research in Obstructive Pulmonary Diseases, Department of Respiratory Medicine, Ghent University Hospital, Ghent, Belgium; 2 Center for Medical Genetics, Ghent University, Ghent, Belgium; 3 University of Groningen, University Medical Center Groningen, Department of Pulmonary Diseases, Groningen, The Netherlands; 4 University of Groningen, University Medical Center Groningen, Department of Pathology and Medical Biology, Groningen, The Netherlands; 5 Department of Pulmonary Diseases, Leiden University Medical Center, Leiden, The Netherlands

## Abstract

Chronic obstructive pulmonary disease (COPD) is characterized by a progressive airflow limitation and is associated with a chronic inflammatory response in both airways and lungs. microRNAs (miRNAs) are often highly conserved between species and have an intricate role within homeostatic conditions and immune responses. Also, miRNAs are dysregulated in smoking-associated diseases. We investigated the miRNA profile of 523 miRNAs by stem-loop RT-qPCR in lung tissue and cell-free bronchoalveolar lavage (BAL) supernatant of mice exposed to air or cigarette smoke (CS) for 4 or 24 weeks. After 24 weeks of CS exposure, 31 miRNAs were differentially expressed in lung tissue and 78 in BAL supernatant. Next, we correlated the miRNA profiling data to inflammation in BAL and lung, obtained by flow cytometry or ELISA. In addition, we surveyed for overlap with newly assessed miRNA profiles in bronchial biopsies and with previously assessed miRNA profiles in lung tissue and induced sputum supernatant of smokers with COPD. Several miRNAs showed concordant differential expression between both species including miR-31*, miR-155, miR-218 and let-7c. Thus, investigating miRNA profiling data in different compartments and both species provided accumulating insights in miRNAs that may be relevant in CS-induced inflammation and the pathogenesis of COPD.

## Introduction

Chronic Obstructive Pulmonary Disease (COPD) is a debilitating respiratory condition characterized by a progressive and irreversible airflow limitation due to an abnormal inflammatory response to inhaled noxious particles or gases^1–3^. The pathology comprises a mixture of small airway obstruction and destruction of lung parenchyma (emphysema); their relative contribution varying between patients and within the lung^4^. The main risk factor for COPD is cigarette smoking. However, only 20% of smokers develop COPD, suggesting that genetic susceptibility or alterations in the epigenetic machinery may be of importance in the development of the disease.

microRNAs (miRNAs), i.e. small non-coding RNAs, are key regulators in diverse biological pathways. One single miRNA can bind to target sequences in multiple mRNAs, typically resulting in mRNA degradation or translational inhibition^5^. By doing so, miRNAs embed a post-transcriptional control within multiple gene signaling cascades. Also, certain miRNAs are critically involved in immune cell development and function. Given this far-reaching influence, it is not surprising that altered miRNA levels contribute to disease pathogenesis^6^.

Ideally, differentially expressed miRNAs in disease versus control can serve as biomarkers of disease initiation/progression or as therapeutic target. In lungs of patients with COPD, we have shown the involvement of down-regulated miRNA-218-5p in recruiting inflammatory cells towards the airways, thereby assisting in the sustained inflammation^7^. In sputum of patients with COPD, down-regulation of let-7c was inversely correlated with soluble TNFR-II, a receptor implicated in COPD pathogenesis^8^.

The expression of miRNAs is described to be altered after cigarette smoke (CS) exposure in lungs of mice and patients with COPD^9–12^. Smoking can potentiate inflammatory processes by affecting the expression of miRNAs that play a key role in immune responses. To obtain full insights in the CS-induced alterations in murine miRNA levels and immune cell populations in the lung, we performed an RT-qPCR-based miRNA profiling in bronchoalveolar lavage (BAL) supernatant and lung tissue of mice that were exposed to air or CS for 4 or 24 weeks, complemented with data on inflammation in BAL and lungs. Moreover, using a translational approach, we checked for overlapping miRNAs in lung tissue between CS-exposed mice and bronchial biopsies of patients with COPD. We also assessed overlap in differentially expressed miRNAs with previously reported studies in patients with COPD. Overall, this information will highlight relevant miRNAs in the CS-induced inflammation.

## Results

### miRNA expression profiling in lung tissue of air- and CS-exposed mice

**Figure 1:**
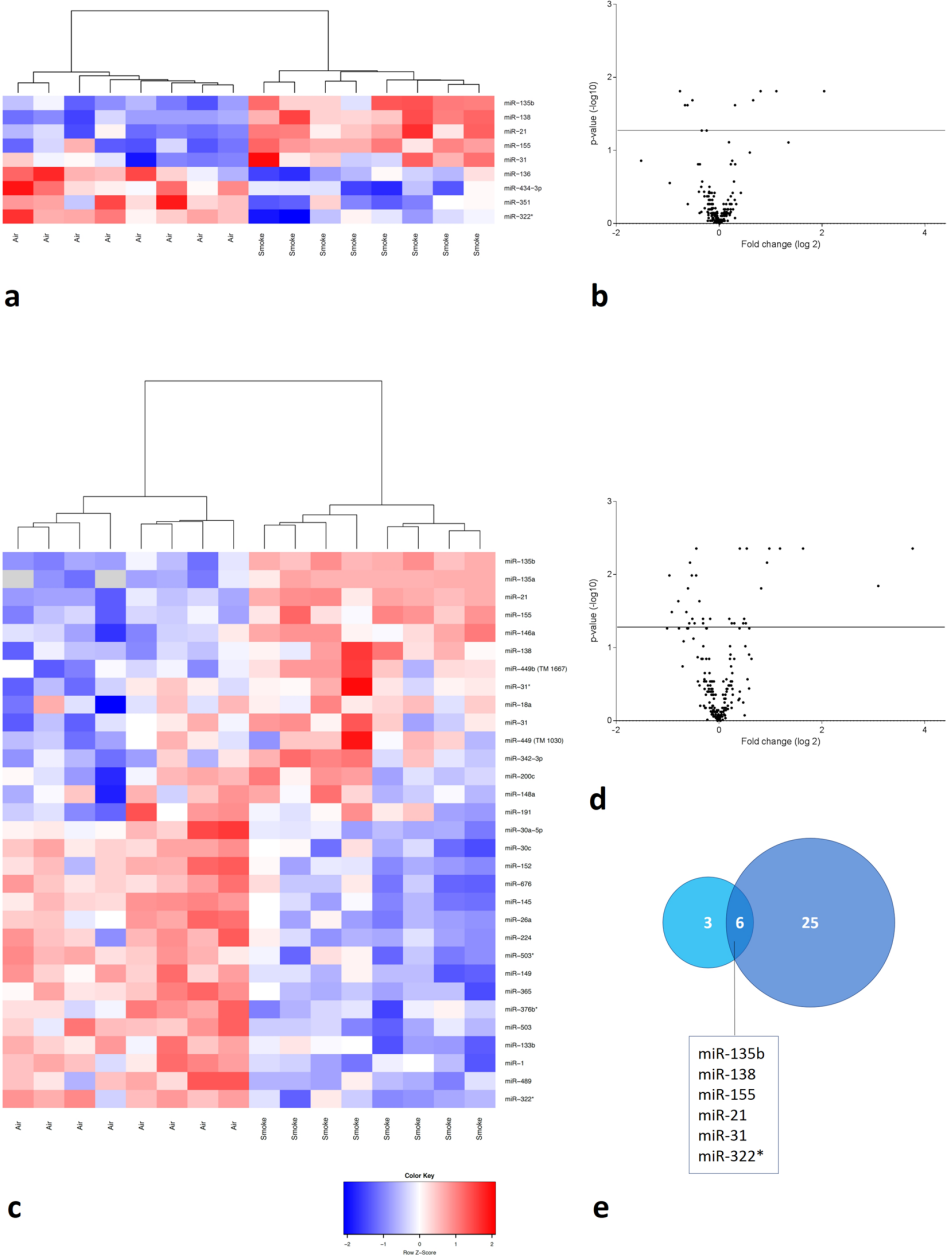
miRNA expression profiling in lung tissue of air- and CS-exposed mice. Volcano plots showing the differential miRNA expression (in fold change on the *x*-axis) and significance level (−log_10_-adjusted p-value on *y*-axis). The detected miRNAs are plotted as black dots. The horizontal line indicates the 0.05 significance level. In the heatmaps, only the significantly differentially expressed miRNAs are represented. These were hierarchically clustered across the air- and smoke-exposed groups. Each row represents a miRNA and each column represents a murine lung sample. The color code indicates the expression level: red = higher expression following CS exposure, blue = lower expression, grey = miRNA was not detected (“NA” values). (**a**) Heatmap showing the differentially expressed miRNAs in murine lung tissue following subacute (4 weeks) air or CS exposure. (**b**) Volcano plot representing the miRNA profiling data following subacute CS exposure compared to air exposure. (**c**) Heatmap showing the differentially expressed miRNAs in murine lung tissue following chronic (24 weeks) air or CS exposure. (**d**) Volcano plot representing the miRNA profiling data following chronic CS exposure versus air exposure. (**e**) Overlap in differentially expressed miRNAs in murine lung tissue between subacute and chronic CS exposure compared to air exposure. Volcano plots with annotated miRNAs can be found in the online supplement (Figure S1).

miRNA profiling was performed on lung tissue of C57BL/6 mice (8 per group), that were exposed to either air or CS for 4 or 24 weeks. Of the 523 miRNAs tested by stem-loop RT-qPCR, 255 miRNAs could be detected. After 4 weeks of CS exposure, 9 miRNAs exhibited differential pulmonary expression compared to the air-exposed mice (4 down-regulated and 5 up-regulated). After 24 weeks of CS exposure, 31 miRNAs showed a significant differential expression of which 16 were down-regulated and 15 were up-regulated. The results are represented in Fig. 1. A list of all differentially expressed miRNAs in lung tissue can be found in Tables 1 and 2 and are annotated on the volcano plots in Figure S1.

**Table 1:** Differentially expressed microRNAs in murine lung tissue following 4 weeks of cigarette smoke exposure.

Up-regulated in CS-exposed mice	miRBase release 21	Fold change	Adjusted p-value
mmu-miR-135b	mmu-miR-135b-5p	4.110	0.015
mmu-miR-138	mmu-miR-138-5p	2.160	0.015
mmu-miR-21	mmu-miR-21a-5p	1.747	0.015
mmu-miR-155	mmu-miR-155-5p	1.580	0.021
mmu-miR-31	mmu-miR-31-5p	1.238	0.024
**Down-regulated in CS-exposed mice**	**miRBase release 21**	**Fold change**	**Adjusted p-value**
mmu-miR-322x	mmu-miR-322-3p	1.695	0.015
mmu-miR-351	mmu-miR-351-5p	1.585	0.024
mmu-miR-434-3p	mmu-miR-434-3p	1.531	0.024
mmu-miR-136	mmu-miR-136-5p	1.531	0.021

First column: annotation of miRNA during microRNA profiling; Second column: annotation of miRNA according to miRBase release 21.

**Table 2:** Differentially expressed microRNAs in murine lung tissue following 24 weeks of cigarette smoke exposure.

Up-regulated in CS-exposed mice	miRBase release 21	Fold change	Adjusted p-value
mmu-miR-135b	mmu-miR-135b-5p	13.593	0.004
mmu-miR-135a	mmu-miR-135a-5p	8.557	0.014
mmu-miR-21	mmu-miR-21a-5p	3.103	0.004
mmu-miR-155	mmu-miR-155-5p	2.274	0.004
mmu-miR-146a	mmu-miR-146a-5p	1.969	0.004
mmu-miR-138	mmu-miR-138-5p	1.908	0.007
mmu-miR-449b	mmu-miR-449c-5p	1.764	0.015
mmu-miR-31x	mmu-miR-31-3p	1.452	0.004
mmu-miR-18a	mmu-miR-18a-5p	1.435	0.046
mmu-miR-31	mmu-miR-31-5p	1.403	0.040
mmu-miR-449	mmu-miR-449a-5p	1.386	0.046
mmu-miR-342-3p	mmu-miR-342-3p	1.322	0.004
mmu-miR-200c	mmu-miR-200c-3p	1.229	0.046
mmu-miR-148a	mmu-miR-148a-3p	1.186	0.046
mmu-miR-191	mmu-miR-191-5p	1.148	0.040
**Down-regulated in CS-exposed mice**	**miRBase release 21**	**Fold change**	**Adjusted p-value**
mmu-miR-322x	mmu-miR-322-3p	1.953	0.010
mmu-miR-489	mmu-miR-489-3p	1.890	0.033
mmu-miR-1	mmu-miR-1a-3p	1.733	0.023
mmu-miR-133b	mmu-miR-133b-3p	1.553	0.033
mmu-miR-503	mmu-miR-503-5p	1.520	0.015
mmu-miR-376bx	mmu-miR-376b-5p	1.493	0.046
mmu-miR-365	mmu-miR-365-3p	1.479	0.007
mmu-miR-149	mmu-miR-149-5p	1.443	0.010
mmu-miR-503x	mmu-miR-503-3p	1.433	0.040
mmu-miR-224	mmu-miR-224-5p	1.393	0.046
mmu-miR-26a	mmu-miR-26a-5p	1.362	0.010
mmu-miR-145	mmu-miR-145a-5p	1.359	0.004
mmu-miR-676	mmu-miR-676-3p	1.302	0.023
mmu-miR-152	mmu-miR-152-3p	1.242	0.040
mmu-miR-30c	mmu-miR-30c-5p	1.241	0.046
mmu-miR-30a-5p	mmu-miR-30a-5p	1.127	0.040

First column: annotation of miRNA during microRNA profiling; Second column: annotation of miRNA according to miRBase release 21.

In lung tissue, all 5 significantly up-regulated miRNAs after 4 weeks of CS exposure, were even more increased following chronic CS exposure, indicating a robust and progressive miRNA signature. On top of the list, miR-135b displayed the highest fold change in lung tissue (Fold change = 13.59, Table 2). From the significantly down-regulated miRNAs following 4 weeks of CS exposure in lung, only miR-322* was still significantly decreased after 24 weeks of CS exposure (Fig. 1e).

### miRNA expression profiling in BAL supernatant of air- and CS-exposed mice

**Figure 2:**
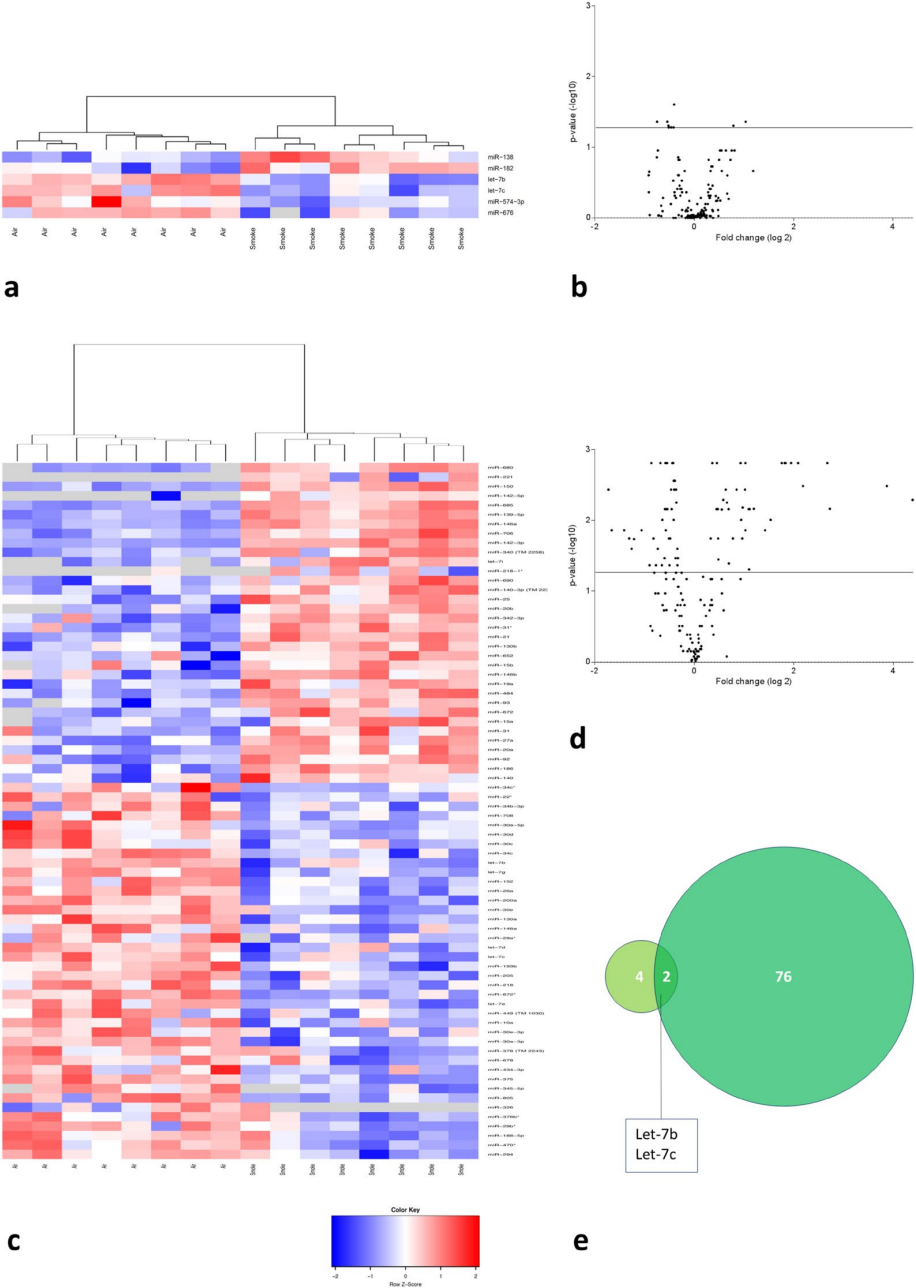
miRNA expression profiling in cell-free BAL supernatant of air- and CS-exposed mice. Volcano plots showing the differential miRNA expression (in fold change on the *x*-axis) and significance level (-log10-adjusted p-value on *y*-axis). The detected miRNAs are plotted as black dots. The horizontal line indicates the 0.05 significance level. In the heatmaps, only the significantly differentially expressed miRNAs are represented. These were hierarchically clustered across the air- and smoke-exposed groups. Each row represents a miRNA and each column represents a murine BAL supernatant sample. The color code indicates the expression level: red = higher expression following CS exposure, blue = lower expression, grey = miRNA was not detected (“NA” values). (**a**) Heatmap showing the differentially expressed miRNAs in murine BAL supernatant following subacute (4 weeks) air or CS exposure. (**b**) Volcano plot representing the miRNA profiling data in BAL supernatant following subacute CS exposure compared to air exposure. (**c**) Heatmap showing the differentially expressed miRNAs in murine BAL supernatant following chronic (24 weeks) air or CS exposure. (**d**) Volcano plot representing the miRNA profiling data in BAL supernatant following chronic CS exposure versus air exposure. (**e**) Overlap in differentially expressed miRNAs in murine BAL supernatant between subacute and chronic CS exposure compared to air exposure. Volcano plots with annotated miRNAs can be found in the online supplement (Figure S2).

miRNA expression profiling was performed on cell-free BAL supernatant of mice exposed to air or CS for 4 or 24 weeks. Of the 523 miRNAs evaluated, 160 miRNAs could be detected in BAL supernatant. After 4 weeks of CS exposure, only 6 miRNAs were significantly differentially expressed (4 down-regulated and 2 up-regulated). However, after 24 weeks of CS smoke exposure, 78 miRNAs exhibited significant differential expression in BAL supernatant of which 40 were down-regulated and 38 were up-regulated. The results are represented in Fig. 2. A list of all differentially expressed miRNAs in BAL supernatant can be found in Tables 3–5 and are annotated on the volcano plots in Figure S2.

**Table 3:** Differentially expressed microRNAs in bronchoalveolar lavage supernatant following 4 weeks of cigarette smoke exposure.

Up-regulated in CS-exposed mice	miRBase release 21	Fold change	Adjusted p-value
mmu-miR-138	mmu-miR-138-5p	2.048	0.044
mmu-miR-182	mmu-miR-182-5p	1.727	0.050
**Down-regulated in CS-exposed mice**	**miRBase release 21**	**Fold change**	**Adjusted p-value**
mmu-miR-676	mmu-miR-676-3p	1.678	0.044
mmu-miR-574-3p	mmu-miR-574-3p	1.458	0.044
mmu-let-7c	mmu-let-7c-5p	1.433	0.050
mmu-let-7b	mmu-let-7b-5p	1.325	0.025

First column: annotation of miRNA during microRNA profiling; Second column: annotation of miRNA according to miRBase release 21.

**Table 4:** Up-regulated microRNAs in bronchoalveolar lavage supernatant following 24 weeks of cigarette smoke exposure.

Up-regulated in CS-exposed mice	miRBase release 21	Fold change	Adjusted p-value
mmu-miR-680	mmu-miR-680	20.924	0.005
mmu-miR-221	mmu-miR-221-3p	14.606	0.003
mmu-miR-297ax	mmu-miR-297a-3p	6.614	0.007
mmu-miR-150	mmu-miR-150-5p	6.382	0.002
mmu-miR-142-5p	mmu-miR-142a-5p	4.554	0.003
mmu-miR-685		4.256	0.002
mmu-miR-139-5p	mmu-miR-139-5p	3.853	0.002
mmu-miR-146a	mmu-miR-146a-5p	3.573	0.002
mmu-miR-706	mmu-miR-706	3.531	0.002
mmu-miR-142-3p	mmu-miR-142a-3p	3.382	0.002
mmu-miR-340	mmu-miR-340-3p	2.900	0.010
mmu-miR-592	mmu-miR-592-5p	2.684	0.014
mmu-let-7ax	mmu-let-7a-1-3p	2.294	0.007
mmu-let-7i	mmu-let-7i-5p	2.165	0.007
mmu-miR-218-1x	mmu-miR-218-1-3p	2.149	0.049
mmu-miR-690	mmu-miR-690	2.143	0.007
mmu-miR-140-3p	mmu-miR-140-3p	2.043	0.002
mmu-miR-9x	mmu-miR-9-3p	2.042	0.014
mmu-miR-25	mmu-miR-25-3p	2.030	0.004
mmu-miR-20b	mmu-miR-20b-5p	1.964	0.007
mmu-miR-342-3p	mmu-miR-342-3p	1.954	0.018
mmu-miR-31x	mmu-miR-31-3p	1.923	0.010
mmu-miR-21	mmu-miR-21a-5p	1.909	0.002
mmu-miR-130b	mmu-miR-130b-3p	1.807	0.004
mmu-miR-652	mmu-miR-652-3p	1.717	0.018
mmu-miR-15b	mmu-miR-15b-5p	1.625	0.041
mmu-miR-146b	mmu-miR-146b-5p	1.619	0.007
mmu-miR-19a	mmu-miR-19a-3p	1.580	0.006
mmu-miR-484	mmu-miR-484	1.550	0.004
mmu-miR-93	mmu-miR-93-5p	1.501	0.005
mmu-miR-672	mmu-miR-672-5p	1.471	0.007
mmu-miR-15a	mmu-miR-15a-5p	1.403	0.036
mmu-miR-31	mmu-miR-31-5p	1.397	0.018
mmu-miR-27a	mmu-miR-27a-3p	1.392	0.007
mmu-miR-20a	mmu-miR-20a-5p	1.373	0.002
mmu-miR-92	mmu-miR-92a-3p	1.365	0.007
mmu-miR-186	mmu-miR-186-5p	1.289	0.002
mmu-miR-140	mmu-miR-140-5p	1.261	0.018

First column: annotation of miRNA during microRNA profiling; Second column: annotation of miRNA according to miRBase release 21.

**Table 5:** Down-regulated microRNAs in bronchoalveolar lavage supernatant following 24 weeks of cigarette smoke exposure.

Down-regulated in CS-exposed mice	miRBase release 21	Fold change	Adjusted p-value
mmu-miR-294	mmu-miR-294-3p	3.289	0.004
mmu-miR-470x	mmu-miR-470-3p	3.155	0.014
mmu-miR-188-5p	mmu-miR-188-5p	2.646	0.014
mmu-miR-29bx	mmu-miR-29b-1-5p	2.469	0.018
mmu-miR-376bx	mmu-miR-376b-5p	2.387	0.025
mmu-miR-326	mmu-miR-326-3p	2.304	0.018
mmu-miR-805		2.079	0.014
mmu-miR-345-5p	mmu-miR-345-5p	1.862	0.043
mmu-miR-375	mmu-miR-375-3p	1.812	0.002
mmu-miR-434-3p	mmu-miR-434-3p	1.757	0.018
mmu-miR-678	mmu-miR-678	1.701	0.043
mmu-miR-378	mmu-miR-378-5p	1.658	0.025
mmu-miR-30a-3p	mmu-miR-30a-3p	1.595	0.002
mmu-miR-30e-3p	mmu-miR-30e-3p	1.565	0.043
mmu-miR-10a	mmu-miR-10a-5p	1.553	0.010
mmu-miR-449	mmu-miR-449a-5p	1.506	0.034
mmu-let-7e	mmu-let-7e-5p	1.499	0.007
mmu-miR-872x	mmu-miR-872-3p	1.488	0.002
mmu-miR-218	mmu-miR-218-5p	1.481	0.014
mmu-miR-205	mmu-miR-205-5p	1.466	0.007
mmu-miR-193b	mmu-miR-193b-3p	1.458	0.002
mmu-let-7c	mmu-let-7c-5p	1.451	0.007
mmu-let-7d	mmu-let-7d-5p	1.420	0.018
mmu-miR-29ax	mmu-miR-29a-5p	1.408	0.043
mmu-miR-148a	mmu-miR-148a-3p	1.393	0.018
mmu-miR-130a	mmu-miR-130a-3p	1.377	0.004
mmu-miR-30e	mmu-miR-30e-5p	1.364	0.007
mmu-miR-200a	mmu-miR-200a-3p	1.353	0.002
mmu-miR-26a	mmu-miR-26a-5p	1.339	0.002
mmu-miR-152	mmu-miR-152-3p	1.328	0.010
mmu-let-7g	mmu-let-7g-5p	1.328	0.003
mmu-let-7b	mmu-let-7b-5p	1.321	0.004
mmu-miR-34c	mmu-miR-34c-5p	1.307	0.003
mmu-miR-30c	mmu-miR-30c-5p	1.295	0.034
mmu-miR-30d	mmu-miR-30d-5p	1.287	0.004
mmu-miR-30a-5p	mmu-miR-30a-5p	1.280	0.006
mmu-miR-708	mmu-miR-708-3p	1.264	0.043
mmu-miR-34b-3p	mmu-miR-34b-3p	1.264	0.025
mmu-miR-22x	mmu-miR-22-5p	1.263	0.043
mmu-miR-34cx	mmu-miR-34c-3p	1.253	0.034

First column: annotation of miRNA during microRNA profiling; Second column: annotation of miRNA according to miRBase release 21.

In BAL supernatant, let-7b and let-7c were significantly reduced after 4 and 24 weeks of CS exposure (Fig. 2e). Moreover, miR-680 showed the highest fold change (Fold change = 20.92, Table 4) following long-term smoking.

### Differentially expressed miRNAs following CS exposure in both lung tissue and BAL supernatant

**Figure 3:**
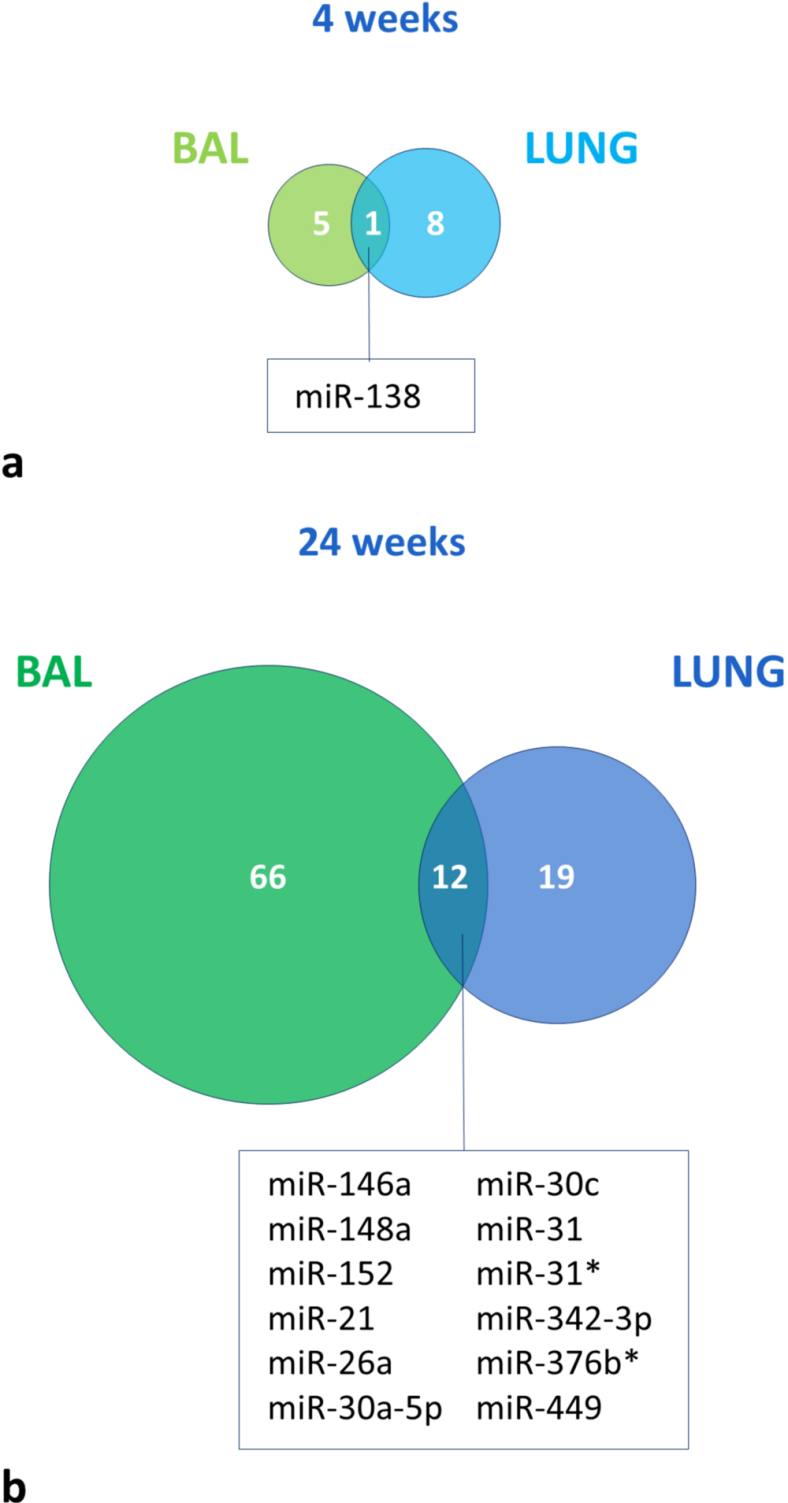
miRNAs that are differentially expressed in both lung tissue and BAL supernatant following (**a**) 4 weeks of CS exposure and following (**b**) 24 weeks of CS exposure.

Following subacute CS exposure, only miR-138 overlaps as being differentially expressed in both lung and BAL supernatant (Fig. 3a). Following chronic CS exposure, 12 miRNAs (miR-146a, miR-148a, miR-152, miR-21, miR-26a, miR-30a-5p, miR-30c, miR-31, miR-31*, miR-342-3p, miR-376b* and miR-449) were differentially expressed in both lung tissue and BAL supernatant of which 10 showed concordant up- or down-regulation. Only miR-449 and miR-148a displayed different expression patterns in the two compartments (Fig. 3b).

Furthermore, a multidimensional scaling plot (Supplementary Figure S3) showed a clear separation between normalized miRNA profiles (only containing miRNAs expressed in both fractions) from BAL supernatant and lung tissue, indicating two compartments with different cellular identity, anatomical structure and organization. miRNA profiles of lung tissue clustered more together than miRNA profiles of BAL supernatant. Moreover, following 24 weeks of CS exposure in BAL, a larger separation in miRNA profiles was noticed compared to lung, underlining the more relative diversity in cell types in BAL following long-term CS exposure. Also, due to this fact and a more activated state of immune cells, a global increase in miRNA abundance was found in BAL supernatant following 24 weeks of CS exposure (Mann-Whitney U test; p-value = 0.007, Supplementary Figure S4).

### Inflammation in lungs and BAL fluid of mice following CS exposure

**Figure 4:**
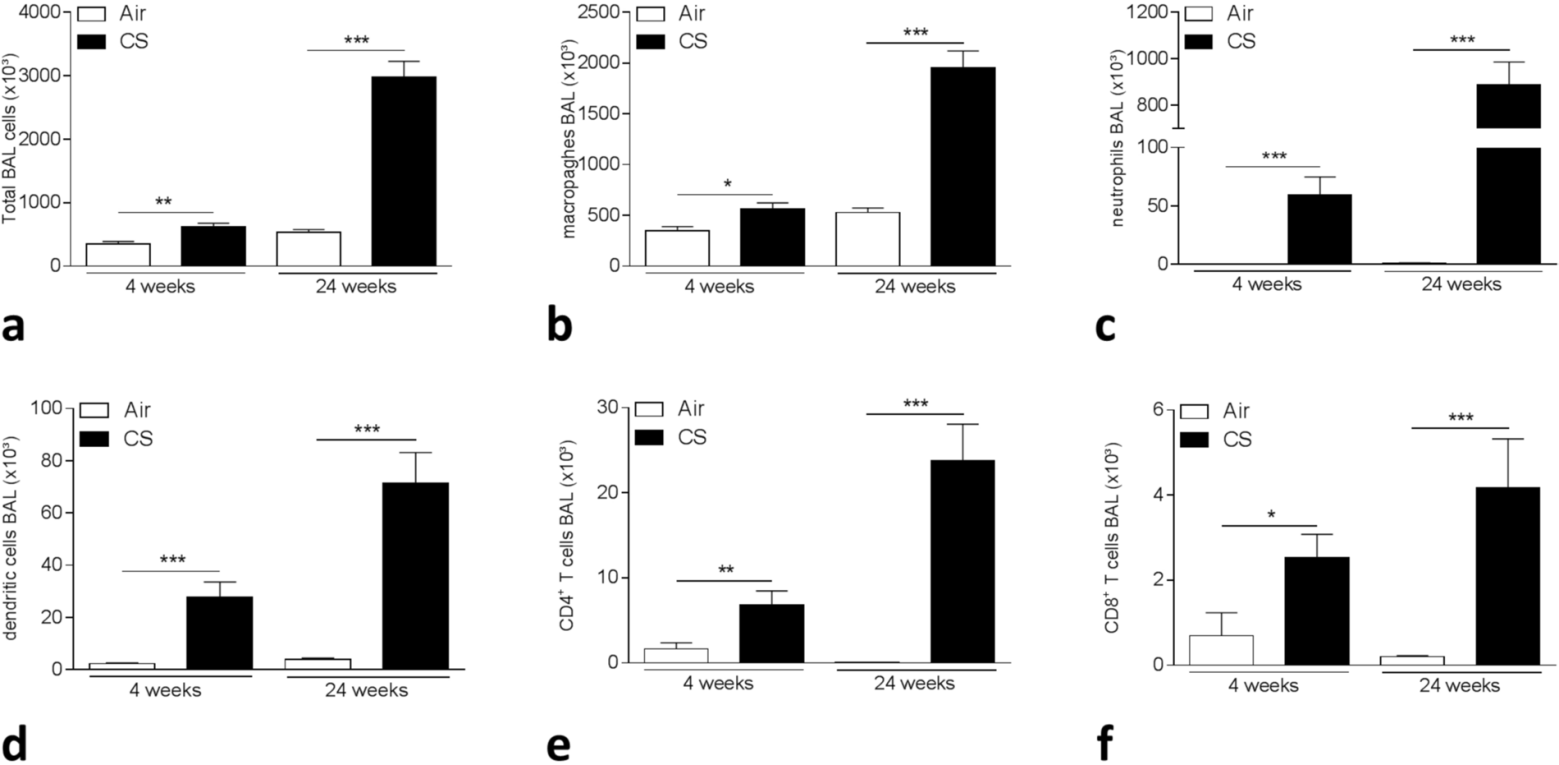
Inflammation in BAL following 4 and 24 weeks of air or CS exposure. (**a**) Total cell numbers in BAL, (**b**) Total macrophages in BAL, (**c**) Total neutrophils in BAL, (**d**) Total dendritic cells (DCs) in BAL, (**e**) Total CD4^+^ T cells in BAL, (**f**) Total CD8^+^ T cells in BAL. *p < 0.05, **p < 0.01, ***p < 0.001.

Four weeks of CS exposure induced an inflammatory response in lung and BAL with recruitment of inflammatory cells and activation of immune signaling. In BAL fluid, there was a significant increase in total cell numbers (p-value = 0.007, Fig. 4a) with more diversity in immune cell subsets such as a significant increase in macrophages (p-value = 0.0104), neutrophils (p-value = 0.00016), dendritic cells (DCs) (p-value = 0.00016) and CD4^+^ (p-value = 0.007) and CD8^+^T lymphocytes (p-value = 0.0104) compared with air-exposed mice (Fig. 4b–f). After 24 weeks of CS exposure, the inflammatory response was severely augmented in BAL with a strong increase in total cell numbers as well as in macrophages, neutrophils, DCs and T lymphocytes (all p-values < 0.001, Fig. 4a–f).

In addition, in lung tissue, the increase in dendritic cells is corresponding to the smoke-induced inflammation and proportionally to subacute or chronic CS exposure compared with air exposure (Supplementary Figure S5a). Also, B cell numbers were augmented following chronic CS exposure (Supplementary Figure S5b).

### Correlation of miRNA expression with inflammatory cell subsets in lung tissue and BAL supernatant

**Figure 5:**
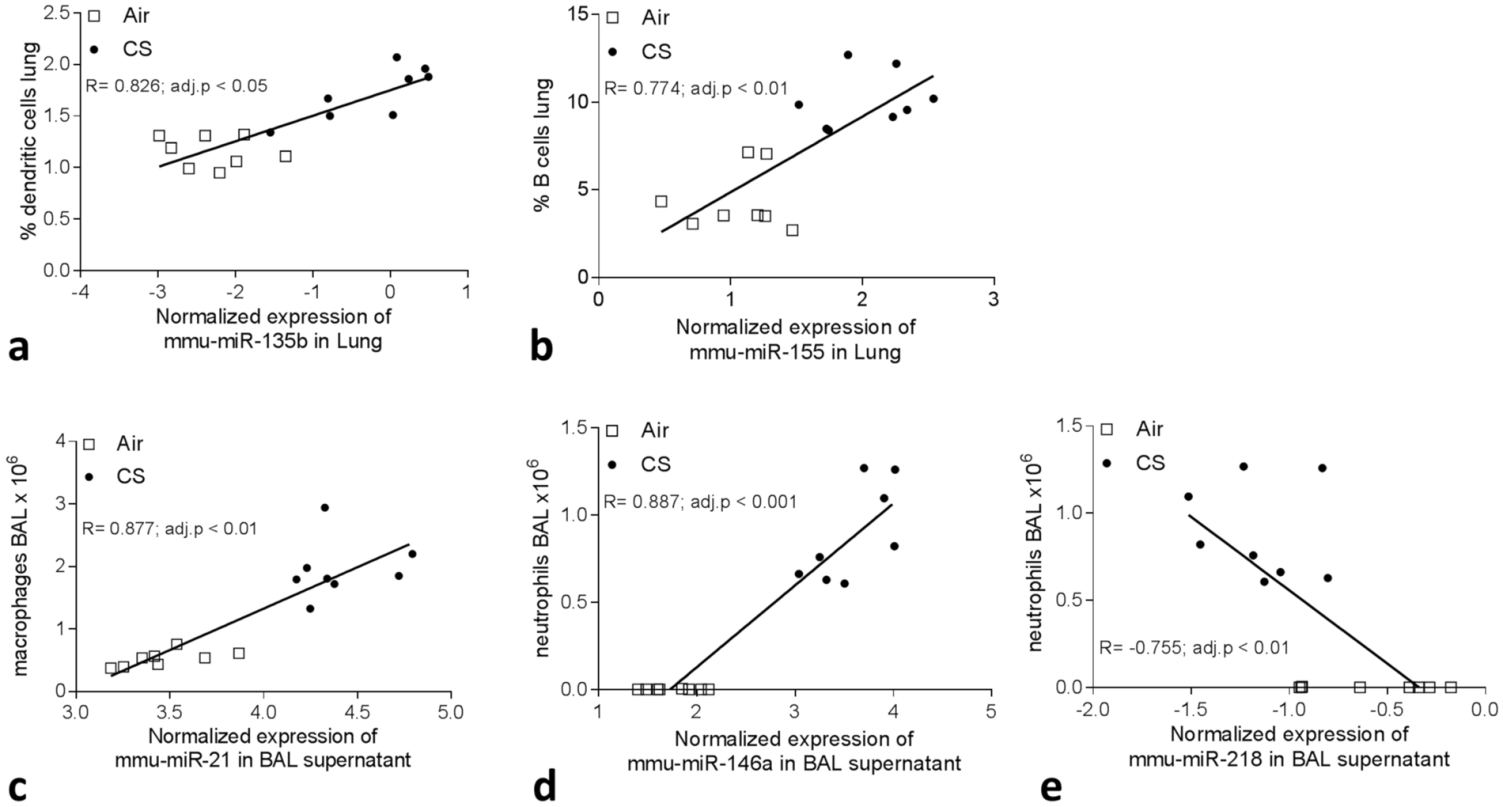
Spearman correlation analyses between the expression of (**a**) miR-135b and % dendritic cells (DCs) in lung following 4 weeks of air or CS exposure. Spearman correlation analysis between the expression of (**b**) miR-155 and % B cells in lung, (**c**) miR-21 and total macrophage numbers in BAL supernatant, (**d**) miR-146a and total neutrophil numbers in BAL supernatant and between (**e**) miR-218 and total neutrophils in BAL supernatant following 24 weeks of air or CS exposure.

To assess whether the change in cell types following CS exposure could be associated with the alteration in miRNA expression, we correlated the miRNA expression with populations of immune cells and levels of inflammatory chemokines. After subacute CS exposure, miR-135b correlated strongly with percentage DCs (adj. p-value = 0.017, Fig. 5a). Following chronic CS exposure, miR-155 correlated significantly with percentage B cells (adj. p-value = 0.0067, Fig. 5b) and miR-152, miR-30a-5p, miR-30c, miR-218 and miR-26a correlated with several immune cell types in lung tissue.

In BAL supernatant, miR-21 correlated significantly with macrophage numbers and MCP-1 (adj. p-value = 0.0014, Fig. 5c), while miR-142-3p, miR-21, miR-146a as well as miR-218 and let-7 family members correlated with neutrophil numbers (Fig. 5d,e). miR-26a and miR-146a correlated with several immune cell types whereas miR-31* correlated significantly with DC numbers (adj. p-value = 0.026).

### Overlap in miRNA expression pattern between chronic CS-exposed mice and patients with COPD

Generally, miRNAs are highly conserved RNA molecules. Therefore, we evaluated a possible overlap in miRNA expression between mice following long-term CS exposure and patients with COPD.

**Figure 6:**
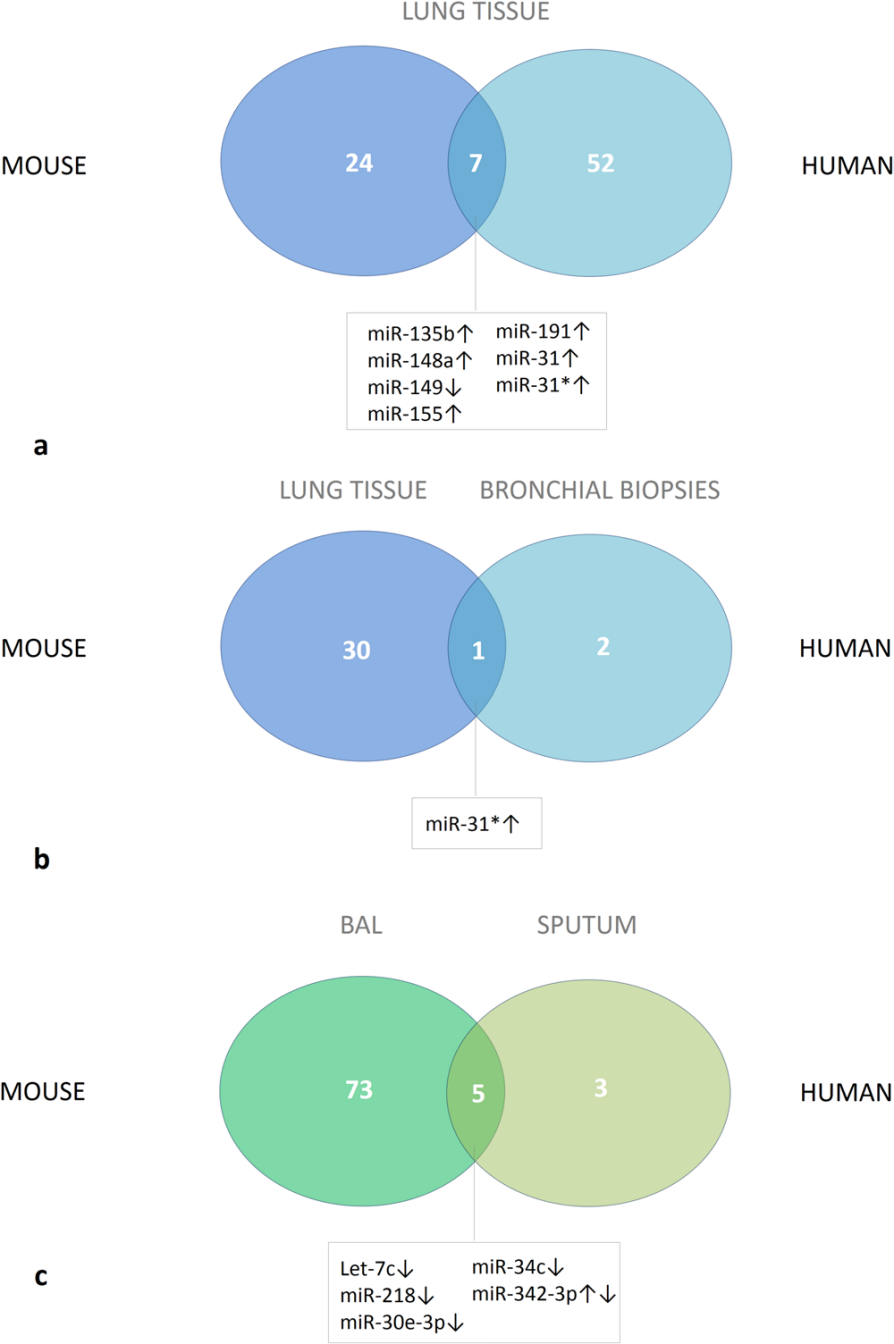
Overlap in miRNA expression between mice following chronic CS exposure and patients with COPD. (**a**) Differential miRNA expression was evaluated in lung tissue of mice that were exposed to 24 weeks of air or CS (n = 8 per group), and in lung tissue of current smoking patients with COPD GOLD II (n = 12) compared to never-smokers (n = 8). (**b**) Differential miRNA expression was evaluated in lung tissue of mice that were exposed to 24 weeks of air or CS, and in bronchial biopsies of current smoking patients with COPD GOLD II-III (n = 42) compared to ex-smoking patients with COPD GOLD II-III (n = 21). (**c**) Differential miRNA expression was evaluated in cell-free bronchoalveolar lavage fluid of mice that were exposed to 24 weeks of air or CS (n = 8 per group), and in induced sputum supernatant of current smoking patients with COPD GOLD I-II (n = 12) compared to never-smokers (n = 10). Numbers of differentially expressed miRNAs are represented as well as the number of overlapping miRNAs. For the overlapping miRNAs, the direction of change in expression following chronic CS exposure /current smoking and having COPD is indicated with arrows. The level of change in miRNA expression can be found in the online supplement (Tables S2–4). COPD: chronic obstructive pulmonary disease, GOLD: Global Initiative for Chronic Obstructive Lung Disease.

First, we evaluated the overlap in lung tissue of mice exposed to CS for 24 weeks and our previously reported miRNA profiling in lung tissue of current smoking COPD patients compared to never-smokers without COPD, all assessed by stem-loop RT-qPCR^7^. Interestingly, miR-135b and miR-155 were significantly up-regulated, both in COPD patients (Adj. p-value (miR-135b) = 0.017; Adj. p-value (miR-155) = 0.0022) versus never-smoking controls and in mice following chronic CS-exposure (Adj. p-value (miR-135b and miR-155) = 0.004) (Fig. 6a) (Table S2).

Second, by integrating data from patients participating in the GLUCOLD study where miRNA expression was newly profiled in bronchial biopsies between current-smoking and ex-smoking patients with moderate to severe COPD, we found an overlap for miR-31* with miRNA profiling in lung tissue of mice chronically exposed to CS compared to air-exposed mice (Fig. 6b), but also with lung tissue of current-smoking COPD patients compared to never-smokers (adj. p-value = 0.012 in GLUCOLD study; adj. p-value = 0.0012 in lung tissue of COPD patients and adj. p-value = 0.004 in mice)^13^ (Table S3). The clinical characteristics of the GLUCOLD study cohort can be found in the online Table S1.

Third, in previously published miRNA profiling data, assessed by the stem-loop RT-qPCR, in induced sputum supernatant of current smoking patients with COPD compared to never-smokers, 8 miRNAs were significantly down-regulated in COPD^8^. Of these 8 miRNA, 5 miRNAs overlapped with the miRNA profile obtained in BAL supernatant of mice chronically exposed to CS compared to air-exposed mice (Fig. 6c). Remarkably, let-7c and miR-218 were significantly reduced (Table S4).

Suplementary Figure S6 shows the sequence conservation between these overlapping human and murine miRNAs.

## Discussion

We have developed a murine model in which CS inhalation – the main etiologic agent in most COPD cases – initiates COPD-like manifestations, enabling us to investigate the CS-induced pathogenesis of COPD^14^. Using these mice, we observed smoke-induced inflammation in BAL and lungs which coincided with changes in miRNA expression in those two compartments. To our knowledge, this is the first time that both lung and BAL supernatant have been thoroughly investigated regarding the miRNA profile.

Several miRNAs were strongly affected by smoke in lung tissue and BAL supernatant. Remarkably, we did not observe an overall down-regulation upon CS exposure in lung as is frequently noted in other miRNA profiling studies^7,15–17^.

By focusing on the overlap between subacute and chronic CS exposure within the same compartment, or the overlap between miRNAs with altered expression levels in BAL and lung, we narrowed the pool of interesting miRNAs down to 18: let-7b, let-7c, miR-135b, miR-138, miR-146a, miR-148a, miR-152, miR-155, miR-21, miR-26a, miR-30a-5p, miR-30c, miR-31, miR-31*, miR-322*, miR-342-3p, miR-376b* and miR-449.

We also assessed whether miRNA profiles clustered together between BAL and lung samples and whether the effect of smoke on the miRNA profile could be distinguished from air-exposed mice (Supplementary Figure S3). The clear separation in miRNA expression between BAL fluid and lung mirrors its different cellular content and structural organization. Moreover, lung tissue was obtained from lavaged mice, meaning that all BAL cells were removed. Also, smoking obviously affected miRNA profiles, both in lung as in BAL supernatant. As expected, a higher diversity in immune cells – often in a more activated state – and a greater amount of cells populate the airways and alveolar spaces following CS exposure, which could lead to a global increase in miRNA abundance in BAL supernatant, favoring certain immune cell-specific miRNAs. To correct for this, we normalized with the mean expression of all miRNAs, rendering a profile relative to this global shift.

CS exposure altered immune cell subsets both in lung and in BAL, as well as it affected miRNA expression levels. We correlated miRNA expression with numbers of inflammatory cells in BAL and lung and found that many of our initially indicated interesting miRNAs were highly correlated. This could mean that altered miRNAs could be implicated in recruitment of these immune cells to the lung or airways, or that they are highly immune cell-specific.

Interestingly, an overlap was observed for miR-135b, miR-148a, miR-149, miR-155, miR-191, miR-31 and miR-31* between lungs of mice chronically exposed to CS and smoking patients with COPD compared to non-smoking controls, suggesting a potential role for these miRNAs in the pathogenesis of COPD^7^. In addition, there was also an overlap for miR-31* between these aforementioned two groups with patients with moderate to severe COPD participating in the GLUCOLD study where the differential miRNA expression was assessed between current- and ex-smoking patients^13^. By surveying the overlap between miRNA profiling data in human sputum supernatant of current smokers with or without airflow limitation compared to never-smokers, and our results in murine BAL supernatant, we found several miRNAs that were concordantly reduced including let-7c, miR-218, miR-26a and miR-449. Although detected in both human sputum and murine BAL supernatant, some miRNAs were expressed in the opposite direction such as miR-146a, miR-342-3p and miR-150^8^.

Regarding miRNA alterations in lung, gradual elevation of miR-155 is generally expected due to its inherent involvement in inflammation since miR-155 modulates both the innate and adaptive immune system^18–21^ and is induced following toll-like receptor (TLR) activation. In agreement with our data, an increase in miR-135b-5p expression was already demonstrated in lungs of mice that were exposed to CS for 4 days and for 18 months, besides the observation that miR-135b-5p is also highly inducible upon challenge of the airways with other noxious particles^22–24^. It is also not surprising that miR-21 is up-regulated in BAL supernatant and in lung tissue. First, miR-21 is suggested to be concordantly expressed between tumor tissue and matched plasma or serum^25,26^. Second, miR-21 is up-regulated in activated immune cell subsets accumulating in the lung upon an inflammatory stimulus, explaining its gradual increase following prolonged smoke exposure. Third, miR-21 is primarily expressed in cells of the macrophage lineage that are already present in the airways without prior CS trigger. Macrophages increase in number following CS exposure but not to the same extent as the newly arrived other immune cells present in BAL, explaining the only moderate increase in miR-21 expression in BAL supernatant.

As expected, miR-21 correlated with macrophage numbers in BAL. Additionally, miR-21 also correlated with MCP-1 protein expression in BAL. It has been shown in a mouse model of abdominal aortic aneurysm that mice exposed to nicotine displayed higher miR-21 levels, which was associated with a reduction in tumor suppressor genes, as well as with an augmentation of inflammatory genes such as IL-6 and MCP-1. Moreover, administering a pre-miR-21 to these mice augmented MCP-1 levels^27^. miR-155, although highly correlated with most immune cell types, showed high association (R_s_ > 0.750) with B cell, CD11b^+^DC and monocyte-derived CD11b^+^DC numbers, highlighting its intricate role in B cell and DC functionality^28,29^. Interestingly, reduced miR-218 expression was negatively correlated (Rs < −0.750) with both neutrophils, B cells, T cells and DC numbers, suggesting that a reduced miR-218 expression could be implicated in directional migration of these cell types towards the inflamed lung^7^. In addition, our results highlight a strong association of miR-31* with active smoking in mice and both cohorts of patients with COPD, as well as with DC subsets. An association of miR-31* with DC numbers is in agreement with a robust increase in both miR-31 transcripts in myeloid dendritic cells upon TLR stimulation in hypoxic conditions^30^.

These results put forward some interesting miRNAs considerably altered following CS exposure both in lung tissue and BAL supernatant of matched murine samples. Correlation of altered miRNA expression with the change in inflammatory profile, suggests a possible implication of these miRNAs in CS-induced inflammation. Evaluating the miRNA expression profile in two different respiratory compartments augments the relevance of our findings, although mechanistic data are lacking. Interestingly, we translated some of our findings to the human situation by discussing the overlap between our murine data and miRNA profiling data in human sputum and lung^7,8^.

In conclusion, we highlight some interesting miRNAs in the CS-induced inflammation in the lung by integrating *in vivo* miRNA profiling data in both BAL supernatant and lung following subacute and chronic CS exposure and *in silico* correlations with inflammatory parameters. Future research will be needed to investigate the exact impact of these miRNAs in the CS-induced inflammation and the pathogenesis of COPD.

## Methods

### Mice

Male C57BL/6 wild-type (WT) mice were purchased from the Jackson Laboratory (Bar Harbor, ME, USA). All mice were kept under a 12 h light-dark cycle in autoclaved cages and bedding, with unlimited access to water and food. The ethics committee for animal experimentation of the faculty of Medicine and Health Sciences (Ghent University) approved all *in vivo* manipulations which were carried out in accordance with guidelines on animal care and welfare. In total, 32 mice were used in this study.

### Smoke exposure

Mice (n = 8 per group) were exposed to air or CS, as described previously^14^. Briefly, mice were exposed whole body to the mainstream tobacco smoke of 5 simultaneously lit 3R4F reference cigarettes (without filter, University of Kentucky, Lexington, KY), 4 times a day with a 30 minutes smoke-free interval. 3R4F reference cigarettes contain 9.4 mg tar, 0.73 mg nicotine, 12.0 mg CO and 11.0 mg total particulate matter, as provided by the supplier. The mice were placed in a plexiglass chamber of 7500 cm^3^, connected to a smoking chamber^14^. Carboxyhemoglobin levels in blood were 8.35 ± 0.47% in CS-exposed mice and 0.65 ± 0.25% in air-exposed mice. The mice were exposed for 5 days per week, for 4 weeks (subacute exposure) or 24 weeks (chronic exposure). An optimal smoke-to-air ratio of 1:6 was maintained. The control groups were exposed to air.

### Bronchoalveolar lavage (BAL)

Via a tracheal cannula, lungs were first lavaged using 3 times 300 µl HBSS (free of Ca2+ and Mg2+ and supplemented with 1% BSA). Supernatant of this fraction was used for ELISA and collected for miRNA profiling. Then, lungs were lavaged using 3 times 1 ml HBSS supplemented with 0.6 mM EDTA. The six lavage fractions were pooled, centrifuged, and the cell pellet was resuspended in 200 µl FACS buffer (PBS supplemented with 1% BSA, 5 mM EDTA and 0.1% sodium azide). Subsequently, total cell counts were obtained using a Bürker chamber and differential cell counts (on at least 400 cells) were performed on cytocentrifuged preparations after May-Grünwald-Giemsa staining. Furthermore, BAL cells were used for flow cytometric analysis.

### Preparation of single cell suspension of lung tissue

Following BAL, the pulmonary and systemic circulation was rinsed with saline, supplemented with 5 mM EDTA. The left lung was used for histology, as described previously^14^. The major lobe of the right lung was taken and thoroughly minced, enzymatically digested and subjected to red blood cell lysis. After passage through a 50 µm cell strainer, cells were counted with a Z2 particle counter (Beckman-Coulter, USA) and left on ice until labeling for further flow cytometric analysis. Another lobe of the right lung was stored for RNA extraction which was later used for miRNA profiling.

### Quantification of inflammation

Flow cytometry was used to enumerate inflammatory cells in BAL fluid and in lung tissue. The analysis was performed on a FACS Calibur (4 weeks exposure experiment) or an LSR Fortessa (24 weeks exposure experiment) (BD Biosciences, San Diego, USA) and data were analyzed with FlowJo software (Tree Star Inc., Ashland, USA). The flow cytometry data in BAL were supplemented with cytospin counts. Chemokines (MCP-1 and KC) were measured in BAL via commercially available ELISA kits (R&D systems).

### RNA extraction

Total RNA from lung tissue and 100 µl of cell-free BAL supernatant was extracted using the miRNeasy mini kit (Qiagen) according to the manufacturer’s instructions. Afterwards, RNA was collected and measured using the Nanodrop 2000 (Thermo Fischer Scientific).

### miRNA profiling on lung tissue of mice that were exposed to air or CS for 4 or 24 weeks

Total RNA, including the small RNA fraction, was reverse transcribed with the miRNA reverse transcription kit (Applied Biosystems, Life Technologies) in combination with a stem-loop Megaplex miRNA primer pool (Applied Biosystems, Life Technologies) consisting of primers for 523 miRNAs and 15 endogenous controls as described previously^31^. After this RT reaction the cDNA was pre-amplified using the TaqMan PreAmp Master Mix and Primer Mix (Applied Biosystems, Life Technologies). This pre-amplification increases the detection sensitivity. The pre-amplified cDNA was diluted 1,600 times. qPCR amplification of 523 mature miRNAs was performed using miRNA TaqMan assays (Applied Biosystems, Life Technologies). The qPCR mixture contained 4 µL of Universal qPCR mastermix, 3 µL of a 1/15 dilution of miRNA TaqMan assay, and 1 µL of diluted preamplified cDNA. All reactions were run on a 7900HT qPCR cycler (Applied Biosystems, Life Technologies) under the following cycling conditions: 10 min at 95 °C followed by 40 cycles of 15 s at 95 °C and 1 min at 60 °C. If the Cq-value was below 32, the miRNAs were considered expressed. The miRNA expression data were normalized using the global mean^32,33^.

Only miRNAs that could be detected (had a Cq-value < 32) in at least 80% of the samples per group were included in our study, resulting in 225 miRNAs out of 523 in mouse lung.

### miRNA profiling on bronchoalveolar lavage supernatant of mice that were exposed to air or CS for 4 or 24 weeks

The same workflow was followed for miRNA profiling on cell-free murine BAL supernatant.

### Human study population

Overlap of murine miRNA profiling data was assessed with 3 different human patient cohorts. First, miRNA profiling was newly performed on bronchial biopsies from current smoking patients with COPD GOLD II-III (n = 42) compared to ex-smoking patients with COPD GOLD II-III (n = 21) participating in the GLUCOLD study^34^. The inclusion criteria for this study required participants without ICS treatment for 6 months and more than 90% of the patients were steroid naïve, i.e. had never used corticosteroids. The GLUCOLD study was approved by the local medical ethics committee of both the Leiden and Groningen University Medical Centers (LUMC and UMCG) and all patients gave their written informed consent. All methods were carried out in accordance with the research guidelines of the Groningen and Leiden Universities^34^. The clinical characteristics of the GLUCOLD study cohort can be found in Table S1.

Second, we also focused on overlapping murine miRNAs with previously published human miRNA profiling data. A miRNA profiling was performed on a) induced sputum supernatant and b) on lung tissue of current smoking patients with COPD compared to never-smokers^7,8^.

### miRNA expression profiling on human bronchial biopsies

Endobronchial biopsies from the GLUCOLD study were immediately snap-frozen and stored at −80 °C. RNA was extracted using the miRNeasy mini kit (Qiagen) according to the manufacturer’s protocol. Purity of RNA fractions was checked on NanoDrop 1000 UV-Vis spectrophotometer and RNA integrity was verified using RNA 6000 Pico Assay RNA chips run on an Agilent 2100 Bio analyzer (Agilent Technologies, Palo Alto, CA). Microarray hybridization was performed at the Boston University Microarray Resource Facility as described in FlashTag™ Biotin HSR Labeling Kit (Affymetrix, Santa Clara, CA, current version available at www.affymetrix.com).

### Annotation of miRNA

Annotation of all differentially expressed miRNAs was updated using miRBase tracker^35^ in Tables 1 and 2 and Supplementary Figures 1 and 2.

### Statistical analysis

Continuous variables were analyzed using non-parametric tests i.e. Mann-Whitney U test when comparing unrelated data using SPSS 24.0 software (SPSS Inc, Chicago, IL, USA). Heatmaps were generated using the heatmap.2 function from the gplots package^36^, where samples were clustered using manhattan distances and the Ward’s method (R statistical programming language, version 3.3.1)^37^. Spearman rank correlation tests between expressed miRNAs from lung and BAL, and a) flow cytometry data in lung and BAL, respectively; b) cytospin data in BAL; c) cytokine/chemokine levels in BAL, were carried out using the cor.test function (R software, version 3.3.1). For the correlation analysis, all miRNAs were included when expressed in at least half of the murine samples. Data were kept when Rs ≥ 0.5 and adjusted p-value ≤ 0.05. The Benjamini-Hochberg procedure was used for multiple testing correction and p-values < 0.05 were considered statistically significant.

### Data availability

The datasets generated during and/or analysed during the current study are available from the corresponding author on reasonable request.

## Electronic supplementary material

Supplemental data
