# Supplementary material for: microRNA profiling in lung tissue and bronchoalveolar lavage of cigarette smoke-exposed mice and in COPD patients: a translational approach
Source: Sci Rep. 2017 Oct 9;7:12871. doi: 10.1038/s41598-017-13265-8 (PMC5634489; doi:10.1038/s41598-017-13265-8)
Supplement: Supplementary file 1 — Supplemental data [file 41598_2017_13265_MOESM1_ESM.pdf]

Online supplement

microRNA profiling in lung tissue and bronchoalveolar lavage of cigarette smoke-exposed mice and in COPD patients: a translational approach

Griet Conickx[1#], Francisco Avila Cobos[2#], Maarten van den Berge[3], Alen Faiz[3], Wim Timens[4], Pieter S. Hiemstra[5], Guy F. Joos[1], Guy G. Brusselle[1], Pieter Mestdagh[2#], Ken R. Bracke[1#]*

[#] Both authors contributed equally to the writing/supervision of the manuscript

FIGURES

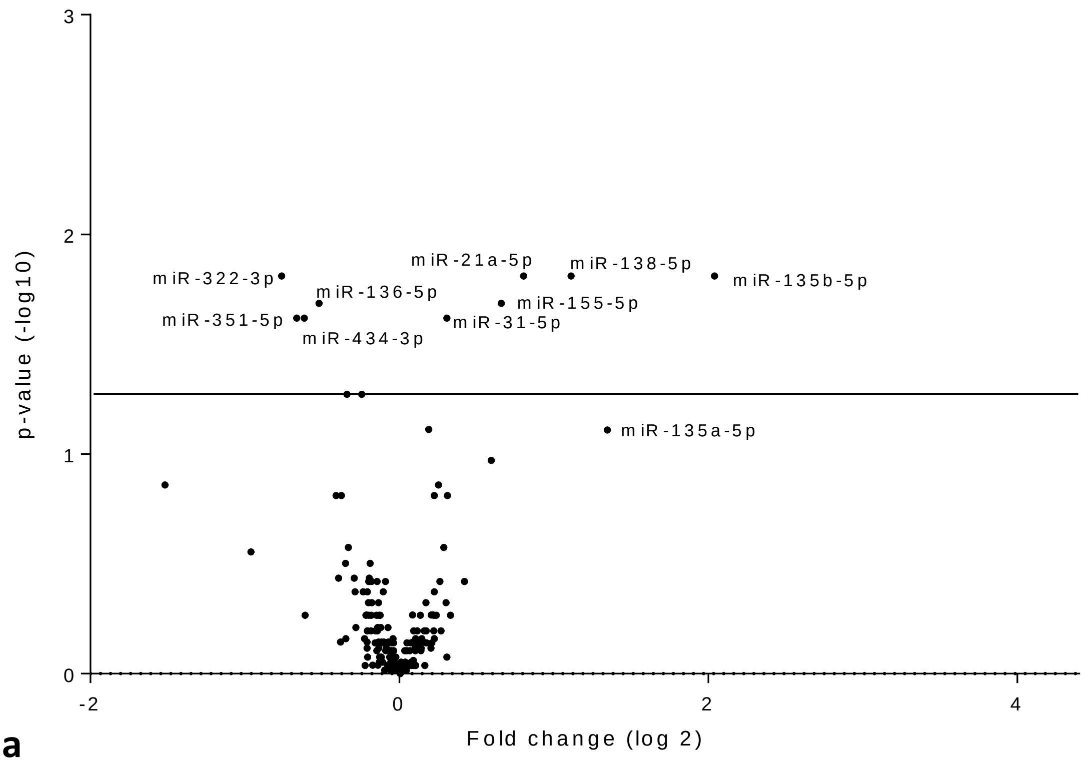

a

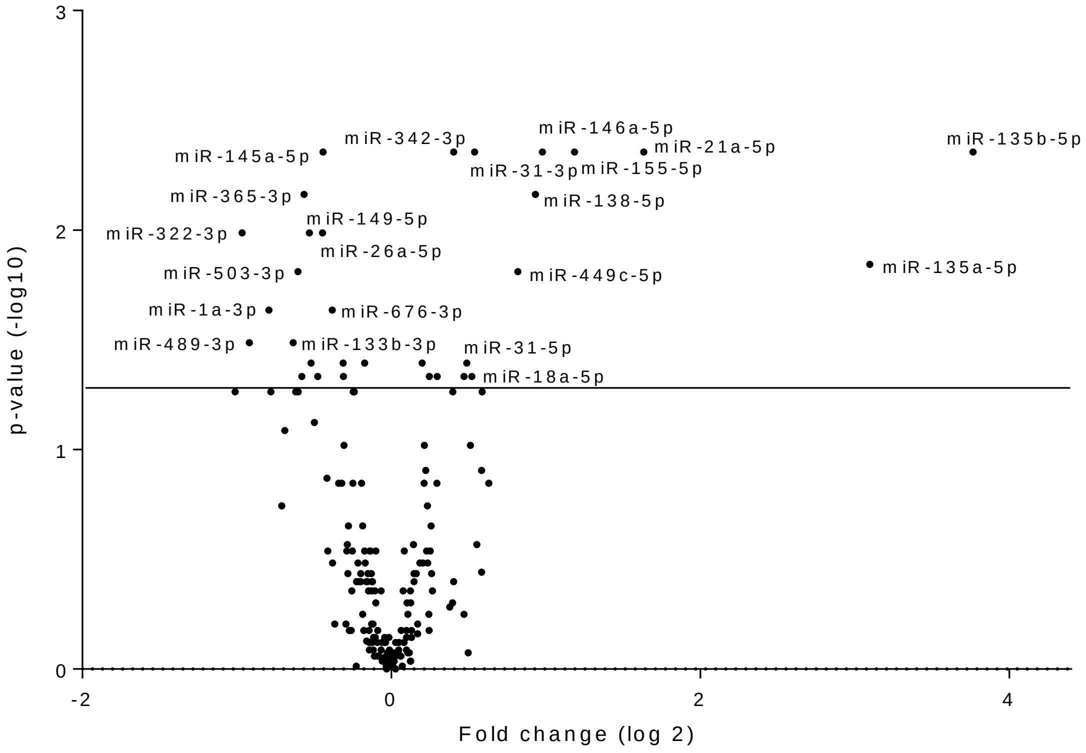

b

Figure S1

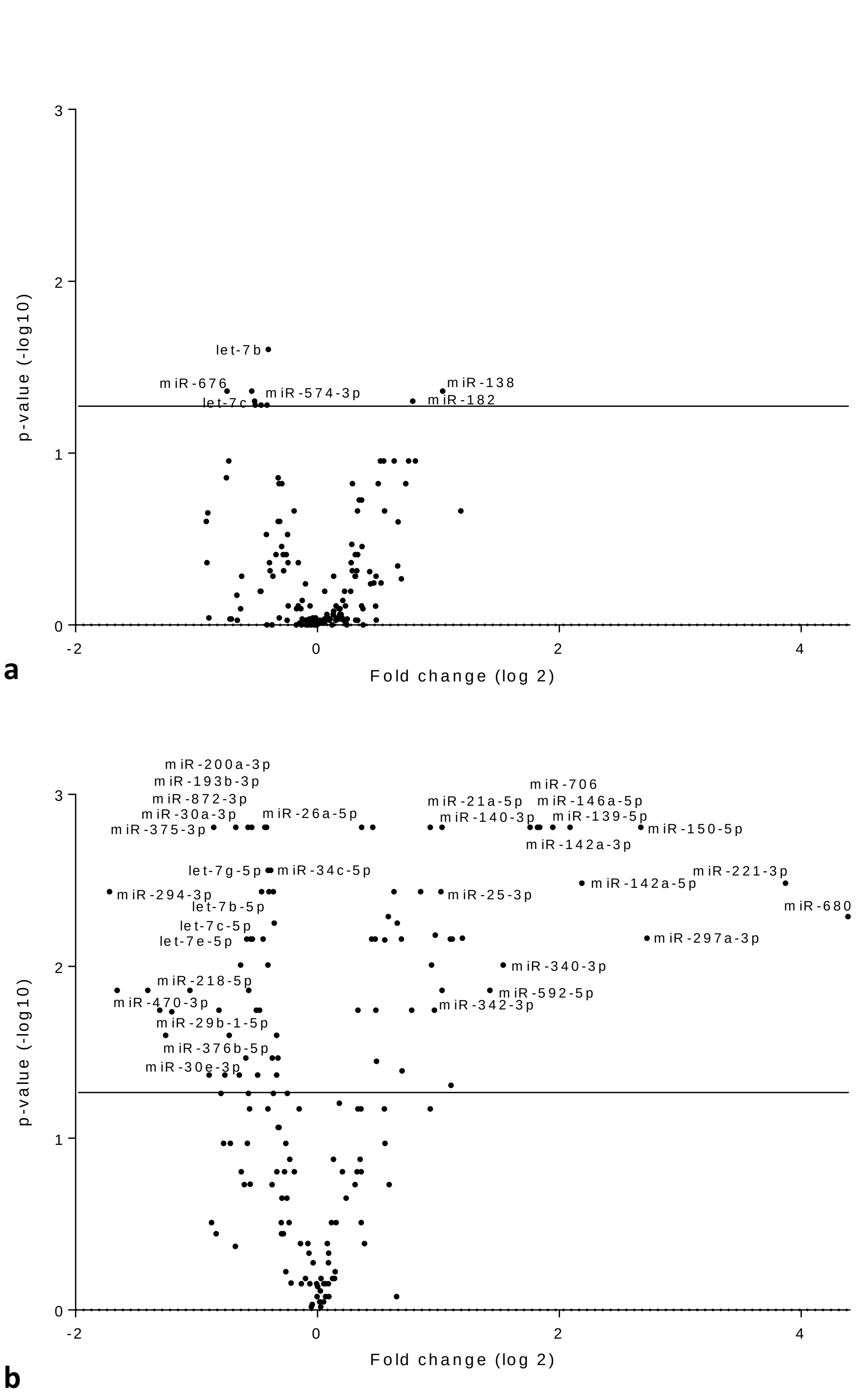
a
b
p-value (-log10)
Fold change (log 2)
let-7b
miR-676
miR-574-3p
let-7c
miR-138
miR-182
miR-200a-3p
miR-193b-3p
miR-872-3p
miR-30a-3p
miR-26a-5p
miR-375-3p
miR-706
miR-21a-5p
miR-146a-5p
miR-140-3p
miR-139-5p
miR-150-5p
miR-142a-3p
let-7g-5p
miR-34c-5p
miR-221-3p
miR-294-3p
miR-142a-5p
miR-25-3p
let-7b-5p
miR-680
let-7c-5p
let-7e-5p
miR-297a-3p
miR-340-3p
miR-218-5p
miR-470-3p
miR-592-5p
miR-342-3p
miR-29b-1-5p
miR-376b-5p
miR-30e-3p

Figure S2

Figure S1. Volcano plots with annotation of differentially expressed miRNAs in lung tissue of cigarette smoke-exposed compared to air-exposed mice. (**a**) 4 weeks of CS/air exposure, (**b**) 24 weeks of CS/Air exposure. Volcano plots showing the differential miRNA expression (in fold change on the *x*-axis) and significance level (-$\log_{10}$-adjusted p-value on *y*-axis). The detected miRNAs are plotted as black dots. The horizontal line indicates the 0.05 significance level. This volcano plot is already presented in Figure 1 (without miRNA annotation).

Figure S2. Volcano plots with annotation of differentially expressed miRNAs in bronchoalveolar lavage supernatant of cigarette smoke-exposed compared to air-exposed mice. (**a**) 4 weeks of CS/air exposure, (**b**) 24 weeks of CS/Air exposure. Volcano plots showing the differential miRNA expression (in fold change on the *x*-axis) and significance level (-$\log_{10}$-adjusted p-value on *y*-axis). The detected miRNAs are plotted as black dots. The horizontal line indicates the 0.05 significance level. This volcano plot is already presented in Figure 2 (without miRNA annotation).

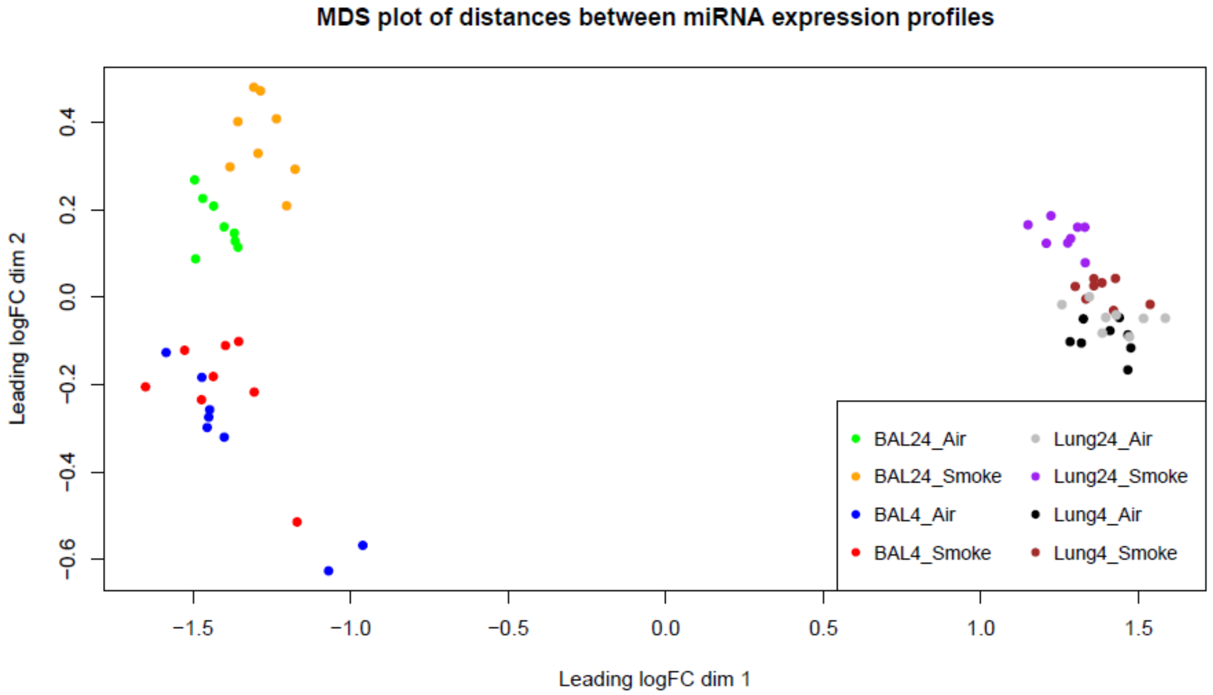

Figure S3. Multidimensional scaling (MDS) plot. This plot visualizes how miRNA profiling experiments cluster together.

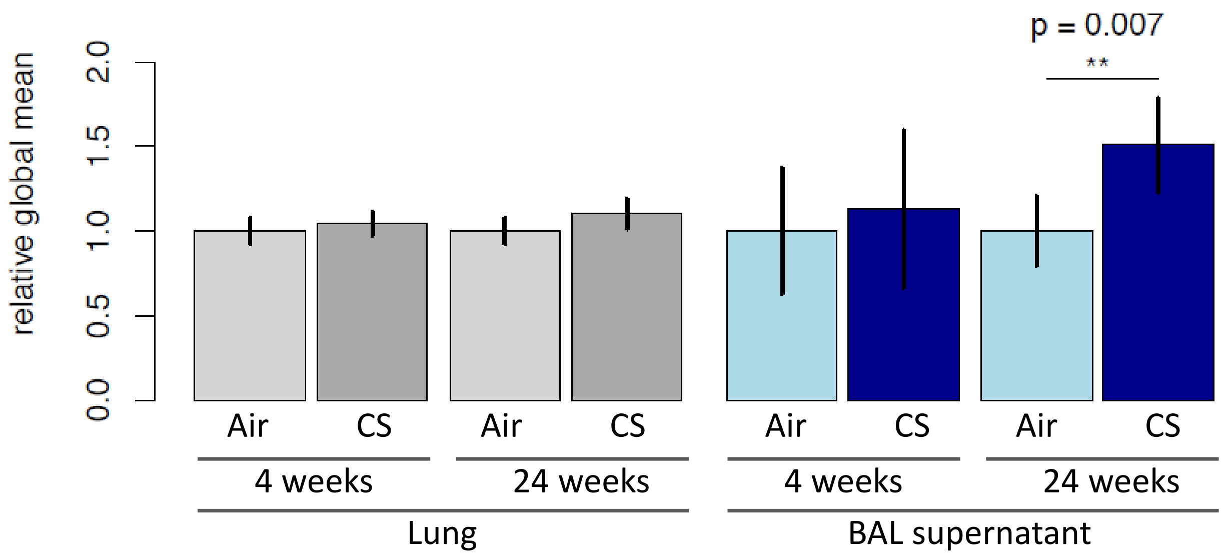

Figure S4. Relative global mean. This graph is obtained by analyzing the global difference in Cq-values between the 2 groups (air versus CS) by performing a Mann-Whitney U test, enabling to detect a possible shift in miRNA expression

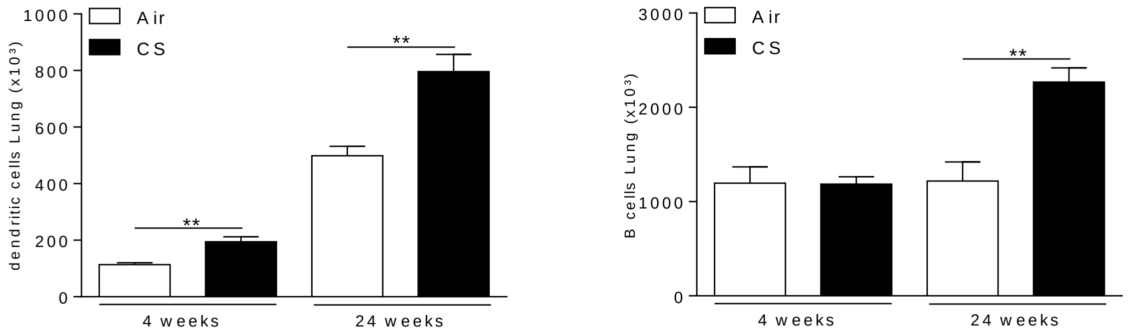

a

b

Figure S5. Inflammation in lung following 4 and 24 weeks of air or CS exposure. (**a**) Total dendritic cells in lung, (**b**) Total B cell numbers in lung following 4 or 24 weeks of air or CS exposure. ** $p < 0.01$

mmu-miR-135b-5p (MIMAT0000612) :	UAUGGCUUUUCAUUCCUAUGUGA
hsa-miR-135b-5p (MIMAT0000758) :	UAUGGCUUUUCAUUCCUAUGUGA
mmu-miR-148a-3p (MIMAT0000516) :	UCAGUGCACUACAGAACUUUGU
hsa-miR-148a-3p (MIMAT0000243) :	UCAGUGCACUACAGAACUUUGU
mmu-miR-149-5p (MIMAT0000159) :	UCUGGCUCCGUGUCUUCACUCCC
hsa-miR-149-5p (MIMAT0000450) :	UCUGGCUCCGUGUCUUCACUCCC
mmu-miR-155b-5p (MIMAT0000165) :	UUAAUGCUAAUUGUGAUAGGGGU
hsa-miR-155b-5p (MIMAT0000646) :	UUAAUGCUAAUCGUGAUAGGGGU
mmu-miR-191-5p (MIMAT0000221) :	CAACGGAAUCCCAAAAGCAGCUG
hsa-miR-191-5p (MIMAT0000440) :	CAACGGAAUCCCAAAAGCAGCUG
mmu-miR-31-5p (MIMAT0000538) :	AGGCAAGAUGCUGGCAUAGCUG
hsa-miR-31-5p (MIMAT0000089) :	AGGCAAGAUGCUGGCAUAGCU
mmu-miR-31-3p (MIMAT0004634) :	UGCUAUGCCAACAUAUUGCCAUC
hsa-miR-31-3p (MIMAT0004504) :	UGCUAUGCCAACAUAUUGCCAU
mmu-let-7c-5p (MIMAT0000523) :	UGAGGUAGUAGGUUGUAUGGUU
hsa-let-7c-5p (MIMAT0000064) :	UGAGGUAGUAGGUUGUAUGGUU
mmu-miR-218-5p (MIMAT0000663) :	UUGUGCUUGAUCUAACCAUGU
hsa-miR-218-5p (MIMAT0000275) :	UUGUGCUUGAUCUAACCAUGU
mmu-miR-30e-3p (MIMAT0000249) :	CUUUCAGUCGGAUGUUUACAGC
hsa-miR-30e-3p (MIMAT0000693) :	CUUUCAGUCGGAUGUUUACAGC
mmu-miR-34c-5p (MIMAT0000381) :	AGGCAGUGUAGUUAGCUGAUUGC
hsa-miR-34c-5p (MIMAT0000686) :	AGGCAGUGUAGUUAGCUGAUUGC
mmu-miR-342-3p (MIMAT0000590) :	UCUCACACAGAAAUCGCACCCGU
hsa-miR-342-3p (MIMAT0000753) :	UCUCACACAGAAAUCGCACCCGU

Figure S6. Sequence conservation of the miRNAs that overlap in Figure 6

TABLES

Table S1. Clinical characteristics of the GLUCOLD study population[1]

	ex-smoker with COPD	current-smoker with COPD
**n**	22	41
**male n (%)**	20 (90.9)	34 (82.9)
**age (years)**	63.36 ± 8.19	58.51 ± 7.97
**FEV_1 % predicted**	60.77 ± 9.96	63.25 ± 10.44

Table S2. Overlap in miRNA expression in murine and human lung tissue following chronic cigarette smoke exposure and in patients with COPD. This table provides extra numeric information regarding Figure 6a.

	MOUSE		HUMAN	
	Fold change	Adj. p-value	Fold change	Adj. p-value
miR-135b	13.593	0.004	1.442	0.017
miR-148a	1.186	0.046	1.285	0.029
miR-149	-1.443	0.010	-1.514	0.024
miR-155	2.274	0.004	1.695	0.002
miR-191	1.148	0.040	1.265	0.034
miR-31	1.403	0.040	2.273	0.049
miR-31*	1.452	0.004	2.743	0.013

Table S3. Overlap in miRNA expression in murine lung tissue following chronic cigarette smoke exposure compared to air exposure and in bronchial biopsies of current smoking patients with COPD compared to ex-smoking patients with COPD. This table provides extra numeric information regarding Figure 6b.

	MOUSE		HUMAN	
	Fold change	Adj. p-value	Fold change	Adj. p-value
miR-31*	1.452	0.004	2.315	0.012

Table S4. Overlap in miRNA expression in murine BAL supernatant following chronic cigarette smoke exposure and in sputum supernatant of current smoking patients with COPD compared to non-smoking controls. This table provides extra numeric information regarding Figure 6c.

	MOUSE		HUMAN	
	Fold change	Adj. p-value	Fold change	Adj. p-value
let-7c	-1.451	0.007	-2.59	<0.001
miR-218	-1.481	0.014	-3.61	<0.001
miR-30e-3p	-1.565	0.043	-2.02	0.001
miR-34c	-1.307	0.003	-3.77	<0.001
miR-342-3p	1.954	0.018	-2.33	<0.001

References

1 Lapperre, T. S. et al. Effect of fluticasone with and without salmeterol on pulmonary outcomes in chronic obstructive pulmonary disease: a randomized trial. Ann Intern Med 151, 517-527 (2009).
